# Characterising infant and young child feeding practices and the consumption of poultry products in rural Tanzania: A mixed methods approach

**DOI:** 10.1111/mcn.12550

**Published:** 2017-11-02

**Authors:** Julia de Bruyn, Brigitte Bagnol, Ian Darnton‐Hill, Wende Maulaga, Peter C. Thomson, Robyn Alders

**Affiliations:** ^1^ School of Life and Environmental Sciences University of Sydney Sydney New South Wales Australia; ^2^ Charles Perkins Centre University of Sydney Sydney New South Wales Australia; ^3^ International Rural Poultry Centre KYEEMA Foundation Brisbane Queensland Australia; ^4^ Department of Anthropology University of Witswatersrand Johannesburg South Africa; ^5^ The Boden Institute of Obesity, Nutrition, Exercise and Eating Disorders University of Sydney Sydney New South Wales Australia; ^6^ Tanzania Veterinary Laboratory Agency Dar es Salaam Tanzania

**Keywords:** breastfeeding, complementary feeding, cultural context, infant and child nutrition, infant feeding decisions, low income countries animal‐source food

## Abstract

Suboptimal breastfeeding practices, early initiation of complementary feeding, and monotonous cereal‐based diets have been implicated as contributors to continuing high rates of child undernutrition in sub‐Saharan Africa. Nutrition‐sensitive interventions, including agricultural programs that increase access to nutrient‐rich vegetables, legumes, and animal‐source foods, have the potential to achieve sustainable improvements in children's diets. In the quest to evaluate the efficacy of such programs in improving growth and development in the first 2 years of life, there is a role for mixed methods research to better understand existing infant and young child feeding practices. This analysis forms part of a longitudinal study assessing the impact of improvements to poultry health and crop production on diets and growth of 503 randomly selected children from eight rural communities in Manyoni District in central Tanzania. Using an explanatory sequential design, the quantitative phase of data collection was conducted between May 2014 and May 2016, comprising six monthly structured questionnaires, four monthly household‐level documentation of chicken and egg consumption, and fortnightly records of children's breastfeeding status. The subsequent qualitative phase involved in‐depth interviews with a subset of 39 mothers in October 2016. Breastfeeding was almost universal (96.8%) and of long duration (mean = 21.7 months, *SD* = 3.6), but early initiation of complementary feeding was also common (74.4%; mean = 4.0 months, *SD* = 1.8), overwhelmingly driven by maternal perceptions of insufficient milk supply (95.0%). Chicken and eggs were infrequently eaten, but close associations between maternal and child consumption patterns (*p* < .001) suggest the potential for strategies that increase household‐level consumption to bring nutritional benefits to young children.

**Acronyms**
ASF: animal‐source foods
HAZ: height‐for‐age *z*‐score
IYCF: infant and young child feeding
WHO: World Health Organization

## 1 INTRODUCTION

With suboptimal breastfeeding estimated to contribute to over 800,000 child deaths annually (Black et al., 2013), exclusive breastfeeding to 6 months of age has been heralded as one of the most effective interventions to prevent child mortality (Jones, Steketee, Black, Bhutta, & Morris, 2003). This is not yet widely practised in many countries, including in Tanzania where the median duration of exclusive breastfeeding at a national level is reported to be 3 months (Ministry of Health, Community Development, Gender, Elderly and Children, Ministry of Health, National Bureau of Statistics, Office of the Chief Government Statistician and ICF, 2016). Perceived insufficient milk supply has been identified as the most common reason for the early initiation of complementary feeding, across diverse socio‐economic, cultural, and geographic settings (Dettwyler & Fishman, 1992; Gussler & Briesemeister, 1980; Sacco, Caulfield, Gittelsohn, & Martínez, 2006). Factors implicated in this phenomenon include infant crying (Dettwyler & Fishman, 1992; Segura‐Millan, Dewey, & Perez‐Escamilla, 1994; Tully & Dewey, 1985), low maternal confidence in breastfeeding ability (Buxton et al., 1991), inadequate breastfeeding knowledge and technique (Hill & Aldag, 1991; Segura‐Millan et al., 1994), the availability and marketing of infant formula (Greiner, van Esterik, & Latham, 1981) and, in some contexts, insufficient contact between mothers and infants (Gussler & Briesemeister, 1980). The need for public health interventions that provide women with information about lactogenesis, interpretation of infant behaviours such as crying, and strategies to respond to common breastfeeding problems has been noted (Arts et al., 2011; Segura‐Millan et al., 1994).

Beyond 6 months, it is recommended that infants receive safe and nutritionally adequate complementary foods, with continued breastfeeding to 2 years of age or above (WHO, 2002). Monotonous cereal‐based diets and infrequent consumption of nutrient‐rich vegetables, legumes, and animal‐source foods (ASF) place many children in low‐ and middle‐income countries at risk of stunting (low height for age) and micronutrient deficiencies. Analysis of national survey data from Tanzania has revealed only 15.9% of breastfed children 6–23 months of age to meet the requirements for minimum dietary diversity, meal frequency, and acceptable diet (Victor, Baines, Agho, & Dibley, 2014). Consumption of ASF has been shown to promote growth, improved cognitive function, physical activity, and health (Black, 2003; Iannotti et al., 2017; Neumann, Murphy, Gewa, Grillenberger, & Bwibo, 2007), yet despite a ratio of one food‐producing animal for every human in Africa (Turk, 2013), inclusion of ASF in local diets remains limited. Programs that promote health and reduce mortality among livestock have the potential to generate income and improve human diets; however, it is important to recognise that the effects of such programs may be unevenly felt across a community, where decisions around income allocation and household diets reflect social, cultural, and economic influences.

This mixed methods study of infant and young child feeding (IYCF) practices is nested within a cluster randomised controlled trial assessing whether community‐based vaccination programs against Newcastle disease in village chickens and improvements to crop diversity, cultivation, and storage practices improve height‐for‐age *z*‐scores (HAZ) in young children in rural Tanzania and Zambia (Alders et al., 2014). Newcastle disease is a viral disease of poultry, responsible for the loss of economic livelihood and a potential source of nutrition in many low‐ and middle‐income countries (Alders, 2014). Periodic outbreaks result in high mortality among free‐ranging chicken flocks and serve as a disincentive for the investment of time or resources in village chickens and a barrier to the consumption of poultry products. In addressing constraints to poultry and crop production, this trial aims to measure the impact of agricultural interventions on the diets and growth of children.

Within the context of this integrated nutrition program, the present study seeks to better understand child feeding practices with the aim of identifying both current barriers and mechanisms for change. Effective behaviour change interventions for nutrition programs in low‐ and middle‐income countries have been identified to have two key determinants: thoughtful formative research to develop and implement interventions and a hypothesis of impact pathways and relevant behaviour outcomes (Fabrizio, van Liere, & Pelto, 2014). Key objectives were to determine the timing of initiation of complementary feeding and weaning, explore reasons for discontinuation of exclusive breastfeeding prior to 6 months, and characterise existing infant and young child diets, with a specific focus on the consumption of poultry products.

**Key messages**

- Maternal perception of insufficient milk was the predominant driver for initiating complementary feeding before 6 months of age.
- Poultry products were infrequently eaten, but longitudinal analyses indicated a close association between dietary patterns of mothers and their young children, and no evidence of gender‐based customs regarding egg consumption by children.
- Participant‐completed pictorial dietary records, providing seasonal data on chicken and egg consumption, were effective in this low‐literacy setting but relied on support from trained Community Assistants.
- Investing time and multi‐disciplinary research skills to conduct mixed methods assessments of diets and child feeding is central to understanding and addressing nutritional challenges.

## 2 METHODS

### 2.1 Study area and population

This paper presents longitudinal findings from a study of 503 children from eight rural villages in Sanza and Majiri Wards, Manyoni District, Singida Region, in the semi‐arid central zone of Tanzania. Project sites were selected in consultation with government partners at national, regional, and district levels, guided by the prevalence of childhood stunting and the absence of existing nutritional interventions. One third (34%) of Tanzanian children under the age of 5 years were reported as stunted in the most recent national survey (i.e., HAZ greater than two standard deviations below the median of the WHO, 2006, reference population), with a regional stunting prevalence of 29% in Singida and 37% in the adjacent Dodoma Region (Ministry of Health, Community Development, Gender, Elderly and Children et al., 2016).

Ninety‐seven per cent of rural households in Tanzania cultivate crops, with combined agricultural activities (crop, livestock, and labour) estimated to generate 70% of income (Covarrubias, Nsiima, & Zezza, 2012). Agriculture in Tanzania is predominantly rain fed and consequently is highly susceptible to adverse weather patterns (Kubik & Maurel, 2016). A unimodal pattern of rainfall is seen in the study area, with long‐term mean annual rainfall of 624 mm and a mean of 49 rain days reported at a district level (Lema & Majule, 2009). Based on daily records from a centrally located rain gauge in each of the two study sites, rainfall during the first of two wet seasons in the period of data collection was particularly poor, with 447 mm (30 rain days) received in Sanza Ward and 275 mm (21 days) in Majiri Ward.

Following a community‐wide census, lists were compiled of households that met the eligibility criteria of including a child under 24 months of age, currently keeping chickens or having expressed an interest in keeping chickens and intending to reside within the area for the duration of the study. Sample size calculation for the cluster randomised controlled trial involving 20 communities (of which this study evaluates findings from eight) was based on an estimated baseline stunting rate of 35% with an aim of reducing this to 25% by the end of the project (i.e., a 10% reduction), giving 80% power to detect this difference as being significant at the two‐sided 5% level, assuming an intracluster correlation coefficient of .014.

**Figure 1:**
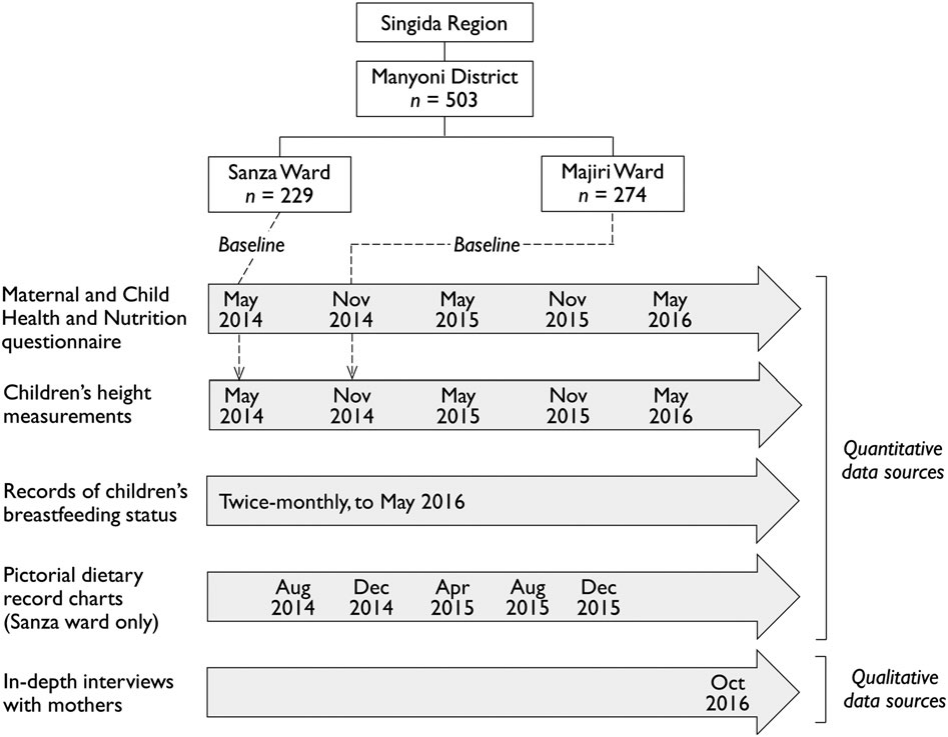
Overview of administrative units in the study area, with the number of enrolled households and the timing of quantitative and qualitative data collection (including the 6‐month delay between baseline data collection in the two wards)

Two‐stage sampling was used to first enrol all eligible households with children under 12 months of age and then enrol additional households with children aged 12–24 months using random selection to give 240 households in Sanza Ward and 280 households in Majiri Ward. Baseline data collection was completed for 229 households in Sanza Ward in May 2014 and 274 households in Majiri Ward in November 2014, as part of the staged implementation within the larger project design, with follow‐up data collected at six monthly intervals to May 2016 (Figure 1).

### 2.2 Quantitative data sources

Male and female enumerators were recruited from the community and trained to administer a coded structured questionnaire to mothers of children enrolled in the study. This questionnaire was developed from the Demographic and Health Survey, applied in Tanzania most recently in 2015 (Ministry of Health, Community Development, Gender, Elderly and Children et al., 2016). Questions covered the timing of initiation of breastfeeding, prelacteal feeding, the timing and nature of complementary feeding, reasons for introduction of complementary foods before 6 months of age, and total duration of breastfeeding. Information was also collected on mothers' participation in formal education, employment and relationship status, water access, and sanitation facilities. Printed survey questions and training sessions were in Swahili, with enumerators encouraged to make use of local languages where appropriate to aid in communication. This questionnaire formed part of the baseline data collection and was applied in an abridged form at six monthly intervals until May 2016, to collate longitudinal information on child feeding practices (Figure 1).

Child length or height measurements were also taken every 6 months to May 2016. Measurements were performed by trained personnel from the Ministry of Health and recorded to the nearest 1 mm using UNICEF portable baby/child length‐height measuring boards. Recumbent length was measured for children up to 24 months of age and standing height for children over 24 months. Where this protocol was not followed, in order to minimise stress to the child and maximise measurement accuracy (6.0% total measurements), a standard adjustment was applied—with standing height approximated to be 7 mm less than recumbent length (WHO, 2006). Child birthdates were verified against health clinic records where possible (80.7%), with some cases where children had not been issued with an official health record, or where records had been misplaced or damaged.

Equal numbers of male and female community representatives (Community Assistants) were employed and trained to visit households on a twice monthly basis for ongoing data collection. Information was recorded on the number of chickens owned and the breastfeeding status of enrolled children within the previous 2 weeks: (a) exclusively breastfed, (b) receiving breast milk and complementary foods, or (c) nonbreastfed. Exclusive breastfeeding was defined as receiving no other food or drink (even water) except breast milk but allowing for oral rehydration solutions and drops or syrups, including vitamins, minerals, and medications (WHO, 2002).

**Figure 2:**
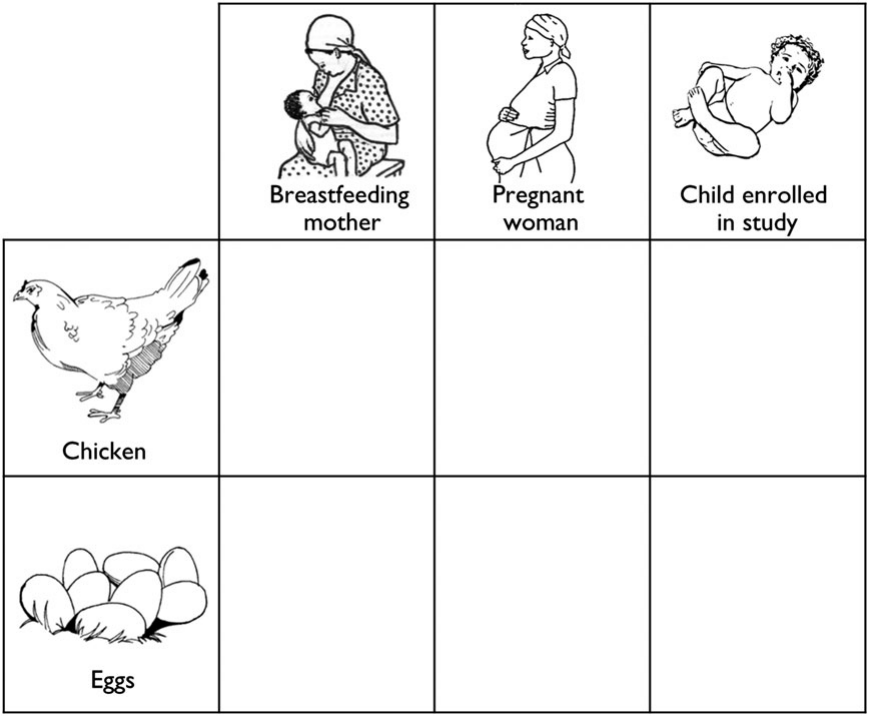
Design of pictorial record chart (with English translations of Swahili text) for completion by a representative of each household, to indicate the consumption of poultry products by children enrolled in the study, and a pregnant or breastfeeding woman within the same household

In Sanza Ward, pictorial record charts were distributed to all enrolled households at four monthly intervals, in the months of August and December in 2014 and April, August, and December in 2015, to document the consumption of poultry products over a period of four consecutive weeks. This research tool was developed for use in communities with low levels of literacy, adapted from an approach used in reproductive health research in Tanzania and Uganda (Francis et al., 2013; Francis et al., 2012) and intended to be able to be used without an understanding of written language. Simple artwork depicting a chicken, eggs, an infant, a pregnant woman, and a breastfeeding mother was presented in a table layout (Figure 2). Prior to each data collection period, the Community Assistants were trained to instruct a representative from each participating household to use a mark to record any meal containing chicken or egg consumed by the enrolled child or by a pregnant or breastfeeding woman in their household (if present). Community Assistants visited each household at the end of each week to review the pictorial charts and assist participants in recording data in any incomplete charts.

### 2.3 Qualitative data sources

The qualitative phase of data collection was conducted in October 2016. Thirty‐nine in‐depth interviews were carried out with a subset of mothers of children enrolled in the longitudinal study. Stratified purposive sampling was used to identify four to six women in each of the eight villages. Eligibility criteria were that women were available on the intended day of interview and willing to engage in discussions for approximately 1 hr. With the aim of achieving diverse representation of households, selection of mothers for interviews was also guided by children's HAZ (both more than two standard deviations below and above the median), marked changes in HAZ over successive measurements (both improving and failing growth patterns), timing of introduction of complementary foods (prior to 6 months, at 6 months and beyond 6 months), chicken ownership and flock size in the previous 24 months (households with no chickens, intermittent and consistent ownership of chickens, and small and larger flocks), and language group (targeting both Gogo and Sukuma households), as determined by prior analysis of questionnaire and anthropometric data.

The majority of interviews were conducted at women's homes, with a smaller number held in a central location in the village at the time that women and their children attended the local health facility. Distances to be travelled to reach women at their home were not a consideration in selection of interviewees. Discussions were conducted predominantly in Swahili with occasional use of the language of the more common group, *Kigogo*, and were led by an English speaker familiar with the study setting, using a semistructured guide with open‐ended questions and facilitated by a translator. For each interview, a Community Assistant was also present to lead introductions and provide additional translation assistance where required. Audio recordings and written notes, predominantly in English, were taken. Questions were based around three main themes: infant and young child feeding, household diets, and poultry keeping. A selection of topics was covered with each interviewee, keeping discussions within the approximate time frame of 1 hr.

### 2.4 Data analysis

Analysis of quantitative data was performed using Genstat software (VSN International, version 18). Descriptive analysis was used to characterise IYCF practices, by ward and in the overall study population, including the timing of initiation of breastfeeding, use of prelacteal fluids, timing and nature of complementary feeding, reasons for introduction of complementary foods prior to 6 months of age, and total duration of breastfeeding. Filter questions within the six monthly questionnaire were designed to restrict data collection on the timing of weaning to events within the prior 6 months but led to missing data when mothers erroneously thought this information had been provided during the previous application of the questionnaire. In these cases and those where mothers were not available to complete the six monthly questionnaire (132 children), children's breastfeeding status was drawn from fortnightly records collected by the Community Assistants.

Demographic characteristics, livestock ownership, and children's height‐for‐age were compared (a) between the two wards and (b) between the interviewed households and others within the study population. Intergroup comparisons were performed using *t* tests and chi‐square tests for continuous and bivariate categorical variables, respectively. Differences were considered significant at *p* < .05.

Descriptive summaries were also compiled using data from pictorial records of chicken and egg consumption to determine the proportion of children and breastfeeding or pregnant women consuming chicken or eggs and mean number of meals containing these food items, over each of the five 4‐week data collection periods. Evaluating children's consumption of chicken and eggs separately, univariable analyses using generalised linear mixed models were initially performed to test associations with child gender, child age, and maternal consumption of chicken or eggs. Geographic and temporal variation was accounted for through the inclusion of ward, village and subvillage locations, and data collection period as random effects. Multivariable models were constructed using variables of suggestive significance (*p* < .1) based on univariable models and backward elimination used to manually remove variables not significant at the 5% level to reach the final models.

Retrospective coding of written interview notes by the primary investigator was used to detect common themes surrounding three broad topics: infant and young child feeding, household diets, and poultry keeping. Thematic analysis was conducted manually, to identify points of consensus and difference among interviewees. Quotations are given in English, derived from translations provided in the context of interviews and later review of audio recordings. Interviewees have been de‐identified, and responses are identified by women's age and household location.

### 2.5 Ethical considerations

Study design, protocols, and research instruments were approved by the National Institute for Medical Research ethics committee (NIMR/HQ/R.8a/Vol.IX/1690) in Tanzania and the University of Sydney Human Research Ethics Committee (2014/209). All participants provided informed consent prior to participating in the study, with assurance of confidentiality, anonymity, voluntary participation, and no adverse effects in case of refusal.

## 3 RESULTS

### 3.1 Characteristics of the study population

An overview of selected demographic characteristics is given in Table 1, for each of the two wards (*n* = 229 in Sanza and *n* = 274 in Majiri), for the overall sample (*n* = 503), and for the subset who participated in the in‐depth interviews (*n* = 39). Significant intergroup differences are indicated. The mean age of children at enrolment was 8.6 months of age. Children within the study sample were significantly older in Sanza Ward (mean age of 9.9 vs. 7.6 months; *p* < .001), as a result of a lower number of households in this area, necessitating the inclusion of more children from the 12‐ to 24‐month category. Approximately one third of children (32.2%) were classified as stunted at the time of first measurement, with a mean HAZ of −1.5.

**Table 1:** Overview of selected demographic characteristics, using baseline questionnaire data: overall, by ward, and in the subset participating in in‐depth interviews

	Sanza Ward	Majiri Ward	Overall	In‐depth interviews
Number of households (*n*)	229	274	503	39
Date of data collection	May 2014	Nov 2014		Oct 2016
Children				
Age at enrolment (months), mean (*SD*)	9.9 (6.1)^a^	7.6 (4.3)^a^	8.6 (5.3)	8.4 (5.6)
Female (%)	55.5	47.4	51.1	35.9
Stunting at baseline (%)	36.8^a^	28.5^a^	32.2	41.7
HAZ at baseline, mean (*SD*)	−1.5 (1.2)	−1.5 (1.1)	−1.5 (1.2)	−1.7 (1.6)
Mothers				
Age at baseline (years), mean (*SD*)	28.5 (7.5)^a^	26.8 (7.5)^a^	27.7 (7.6)	28.9 (7.7)
No formal education (%)	22.8^a^	40.6^a^	32.5	39.5
Households				
Female headed (%)	30.2^a^	16.4^a^	22.7	15.8
Number of members, mean (*SD*)	5.4 (1.9)	5.5 (2.6)	5.4 (2.3)	5.8 (2.4)
Language group (%)				
Gogo	78.2^a^	74.8^a^	76.3	76.9
Sukuma	6.1^a^	14.6^a^	10.7^b^	23.1^b^
Other	4.4	2.6	3.4	0.0
Not specified	11.4^a^	8.0^a^	9.5^b^	0.0^b^
Livestock ownership at baseline (%)				
Chickens	51.1	46.8	48.8^b^	65.8^b^
Goats and sheep	27.1^a^	47.8^a^	38.3	36.8
Cattle	26.7^a^	36.2^a^	31.8^b^	47.4^b^

*Note*. *SD* = standard deviation; HAZ = height‐for‐age *z*‐score; Significant differences (*p* < .05) are indicated as follows:

a Between the two wards.

b Between the overall sample and the subset participating in in‐depth interviews.

Low levels of formal education were seen across the study population, with 32.5% of mothers never having attended school and significant variation between the two wards (22.8% in Sanza vs. 40.6% in Majiri; *p* < .001). Fewer female‐headed households (16.4% vs. 30.2%; *p* < .001), higher levels of small ruminant ownership (47.8% vs. 27.1%; *p* < .001), and greater representation of the Sukuma language group (14.6% vs. 6.1%; *p* = .003) were also seen among the Majiri participants compared with those from Sanza. Among those participating in in‐depth interviews, there was a significantly higher proportion of women from households identifying as Sukuma and from households keeping chickens, as part of a conscious effort to explore differences in IYCF practices between language groups and potential contributions of chickens to household diets.

### 3.2 Timing of breastfeeding and complementary feeding

Approximately two thirds of children were born in a health facility (67.5%), and among those born at home 16.2% were recorded as having been weighed at a health facility within an hour of birth (Table 2). Of 503 children, the vast majority (96.8%) were reported to have been breastfed for any period of time. Breastfeeding was initiated within 1 hr of birth for 77.5% of infants and within 1 day for 93.9%. Mothers of 40.7% of children indicated that fluids other than breast milk had been given to their child in the first 3 days post‐partum, before breast milk production had fully commenced. Water with sugar was the predominant fluid given in these circumstances (28.5% infants), substantially more common than non‐human milk (2.4%) or plain water (1.2%).

**Table 2:** Childbirth, breastfeeding, and early complementary feeding practices of enrolled children in the overall study population and by ward, compiled from six monthly questionnaire responses and fortnightly household visits to record children's breastfeeding status

	Sanza Ward	Majiri Ward	Overall
Enrolled children (*n*)	229	274	503
Delivered by caesarean section (%)	3.5	7.4	5.6
Delivered at home (%)	35.7	29.8	32.5
Weighed at health facility within 1 hr of delivery (%)	27.2	5.1	16.2
Ever breastfed (%)	94.7	98.5	96.8
Breastfeeding initiated within 1 hr (%)	67.5	85.2	77.5
Breastfeeding initiated within 24 hr (%)	88.5	98.5	93.9
Prelacteal feeding (%)	46.5	35.8	40.7
Water with sugar (%)	29.4	27.8	28.5
Water with sugar and salt (%)	13.2	3.7	8.0
Tea (%)	1.8	3.3	2.6
Milk other than human breast milk (%)	2.6	2.2	2.4
Plain water (%)	1.3	1.1	1.2
Age in months at initiation of complementary feeding			
Mean (*SD*)	4.2 (1.9)	3.8 (1.7)	4.0 (1.8)
Range	1–11	1–9	1–11
Exclusively breastfed until 6 months (%)	29.8	22.1	25.6
Reasons for early initiation of complementary feeding (%)			
Insufficient breast milk	89.7	98.0	95.0
Child refused breast milk	6.0	0.5	2.5
Maternal illness	3.4	1.0	1.9
Separation of mother and child	0.9	0.5	0.6
Items commonly added to porridge during early complementary feeding			
Sugar	77.8	86.9	83.3
Nuts or beans	30.4	56.3	46.0
Baobab fruit	12.6	17.0	15.2
Cow's milk	8.1	12.6	10.9
Oil	1.5	1.5	1.5
Egg	1.5	1.0	1.2
Meat	0.0	0.5	0.3
Age in months at weaning			
Mean (*SD*)	22.0 (3.5)	21.3 (3.6)	21.7 (3.6)
Range	11–36	11–29	11–36
Breastfeeding continued until 24 months (%)	38.4	30.5	34.5

*Note*. *SD* = standard deviation.

Questionnaire data indicated the mean age for initiation of complementary feeding to be 4 months (range of 1–11 months) and exclusive breastfeeding to 6 months of age to be practised by 25.6% of mothers. Among interviewed mothers, despite the purposive selection of interviewees introducing bias and resulting in an increased proportion of mothers of stunted children, similar timing of the introduction of complementary foods was reported. Of 28 interviewees with whom the topic was discussed, 18 reported having introduced liquids or foods other than breast milk before 6 months of age (mean age of 3.8 months, range of 2 weeks to 8 months). Two women attested that they did not know of any mothers within their community who had sustained exclusive breastfeeding to 6 months.

In the overall study population, the mean age of weaning was 21.7 months (range of 11–36 months), with 34.5% of mothers meeting WHO recommendations for continued breastfeeding to 24 months of age or above.

### 3.3 Reasons for early initiation of complementary feeding

Among the overall study population, reasons for early introduction of complementary foods were sought through a semistructured question. Of the mothers who reported introducing foods or fluids before 6 months of age (*n* = 374), 95% selected the response of “insufficient breast milk” as the primary reason for their decision from five listed responses read aloud by enumerators (which also included a subsequent pregnancy, child refusal to feed, maternal illness, or separation of mother and child)—with the opportunity for unlisted responses to be recorded. In Sanza Ward, where insufficient breast milk was nominated by a significantly lower proportion of mothers than in Majiri Ward (89.7% cf. 98.0%; *p* = .007), infants' refusal to breastfeed was the second most common reason given (6.0%). More specific observations motivating a change in feeding practices, such as infant crying or weight loss, were not among listed options and were not nominated by any questionnaire respondents.

**Figure 3:**
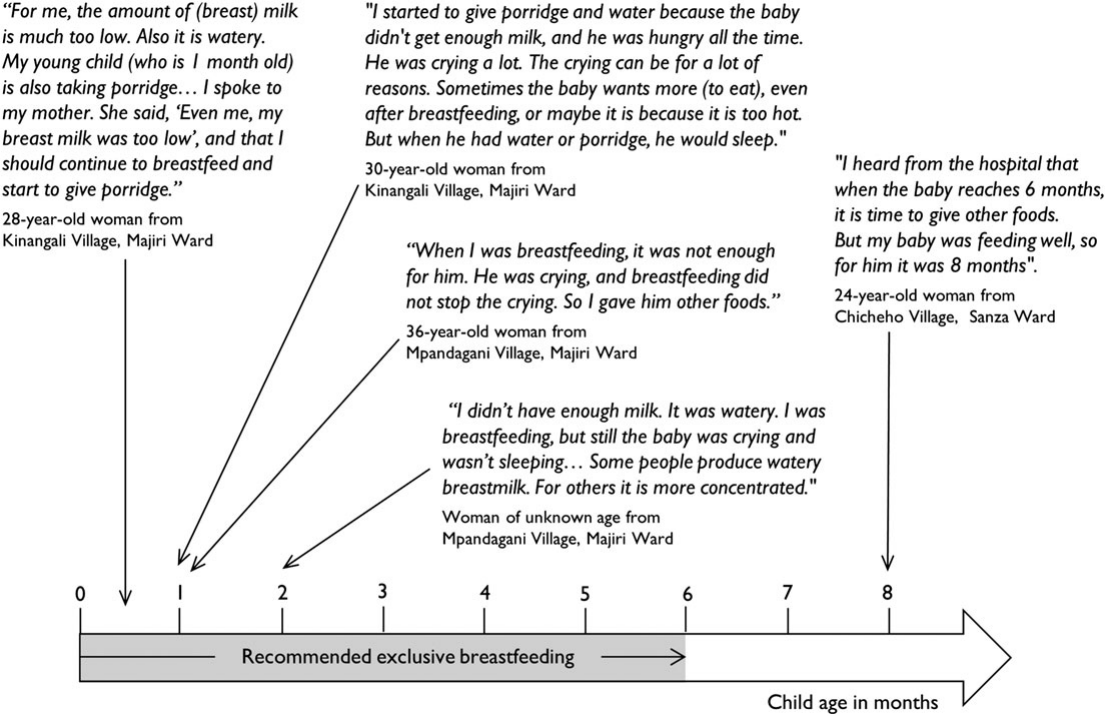
Mothers' experiences of breastfeeding, according to the age of their child at initiation of complementary feeding (qualitative data collected through in‐depth interviews)

In comparison, during interviews, 14 women cited children crying as a reason to introduce other foods, nine identified their milk supply to be insufficient in quantity, and five described the consistency of breast milk as being too watery. Reasons for crying were acknowledged to be many and varied, but a common response to persistent and protracted crying—including after breastfeeding and overnight—was to offer liquids and foods other than breast milk (see Figure 3). Many interviewed mothers indicated their awareness of the recommendation of exclusive breastfeeding to 6 months, with one conveying a firmness in the delivery of this guideline by health personnel: “When you go to the hospital, they tell you that you must breastfeed for six months, in strong voices” (24‐year‐old woman from Chicheho Village, Sanza Ward).

Two women described an association between their child feeding from a particular breast and vomiting, in the absence of any maternal symptoms in this breast. One of these women reported having consulted a nurse at the local health facility, who advised her to persist with feeding from the other breast but said she had opted to initiate complementary feeding at 5 months because she felt that the child was not receiving enough milk. No other women reported having sought advice from health staff about difficulties associated with breastfeeding, although it was indicated that such an opportunity would exist during monthly weight checks for infants. Interviewees more commonly reported discussing breastfeeding difficulties with their mother, mother‐in‐law or, in one case, husband.

### 3.4 Nature of complementary foods

Of 341 infants reported in the questionnaire to have received solid or semi‐solid foods prior to 6 months of age, all were given cereal‐based porridges. Among this subset, a majority (83.3%) of mothers reported sugar to have been added to infants' porridge during the early phase of complementary feeding (Table 2). “Nuts or beans” were reported as being added to porridge for 46.0% of infants, baobab fruit powder for 15.2%, and cow's milk for 10.9%. Other ASF were only mentioned by a small number of mothers, with eggs recorded as being added to porridge for 1.2% of infants and meat for 0.3%. A failure to define the regularity of consumption in this section of the questionnaire may have led to an over‐reporting of food items added to porridge on an infrequent or occasional basis, for reasons such as economic constraints or seasonal availability.

It is possible that baobab fruit, which was not among the listed food items but was recorded in the “additional items” category, was under‐reported in questionnaire responses, depending on enumerators' proficiency in probing for nonlisted food items. Several participants in in‐depth interviews described adding baobab fruit and groundnuts to their young child's porridge—in accordance with questionnaire data—however, further questioning revealed the former to depend on seasonal availability (with most fruit being consumed or sold within a short period following harvest) and the latter to be infrequent due to limited groundnut production in the area.

In‐depth interview findings confirmed a limited range of foods to be offered to children in the initial phases of complementary feeding. Nine women reported water with sugar added to have been the first item other than breast milk offered to their child, particularly when complementary feeding was initiated before 3 months of age. One mother described preparing a watery porridge for her 1‐month‐old twins, passing it through a sieve to remove any solid fragments. Fifteen mothers indicated a soft maize or sorghum porridge with sugar to have been the initial complementary food, and four women (all from cattle‐owning households) reported giving cow's milk.

Moderate variation was evident in mothers' approaches to continued complementary feeding, with no clear association between the age of initiating complementary feeding and the timing of introduction of specific foods. In some cases, children who received soft porridges at an early age started eating *ugali* (a stiff maize‐ or sorghum‐based porridge, the predominant staple dish of the area) and its common accompaniment of green leafy vegetable (typically from noncultivated plant sources) sooner than those adhering to the recommended 6 months of exclusive breastfeeding, who might continue on sweetened porridges until 1 year of age. In other cases, mothers reported introducing both soft porridge and more solid household foods simultaneously, or in quick succession, from 6 months of age.

Three women explained that meat and fish would be introduced later than vegetables and legumes, with one mother mentioning 12 months of age and two suggesting 18 months as an appropriate time. Household consumption of meat and fish among the study population was variable but largely infrequent (see Table 3), suggesting the timing of introduction to be based not only on conscious decisions about a child's readiness for these foods but on their frequency of consumption within the wider household. Although egg consumption was also documented as being very low across the study population (Tables 3 and 4), five women made reference to a specific custom prohibiting their consumption by uncircumcised male children.

**Table 3:** Consumption frequency of selected food items, with associated reasons for and barriers to consumption, compiled from in‐depth interviews with mothers. Findings are presented alongside corresponding food group‐based recommendations for Tanzania

Food group‐based recommendations for adults in Tanzania (TFNC, 2011)	Food item discussed with interviewees	Reported consumption frequency	Reported reasons to consume	Reported barriers to consumption
Animal‐source foods and legumes 2–3 servings per day One serving is equal to A single egg A palm‐sized piece of meat or fish 250 ml milk Half a cup of cooked beans	Eggs	Infrequent consumption by majority of interviewed households (from “never” to “occasionally”). Three women reported eggs to be eaten on a regular basis, every 1–2 weeks. One mother said eggs would be given preferentially to children and particularly to young children.	Availability. A common circumstance for consuming eggs would be when a hen has died or abandoned her eggs or has laid more than she might be able to raise as chicks. One woman described having added egg to her child's porridge to improve her growth, and two others mentioned eggs as a beneficial food for children.	Not owning chickens or owning a small number only. No interviewees mentioned buying eggs to eat. Most women emphasised the need to keep eggs for hatching. (“If you eat eggs, where will you get chickens?”). There was one mention of the significance of language group, with the suggestion that it is not customary for Sukuma families to eat eggs.
	Chicken meat	Substantial variation between households. Only four women indicated chickens to be slaughtered with any regularity, ranging from once per month to three times per week. For others, consumption was mostly associated with special occasions.	Large flock size and the ease of slaughtering chickens at home were cited by those consuming chickens on a regular basis. Visiting guests and special occasions (e.g., public holidays or weddings) were common reasons to eat chicken. Two women said chickens would be consumed at times of vegetable scarcity. Two reported chickens would only be eaten if they died of disease.	Decision‐making on the consumption or sale of chickens was reported to commonly involve the male household head. An emphasis on the need to retain chickens for sale in times of need was a common barrier to more frequent consumption. One woman from a large household indicated the number of chickens required to feed all household members to be a deterrent.
	Other meat or fish	Marked variation between households. Meat or fish were reported to be eaten three times per week by three interviewees and once per week by two. For the majority, consumption was much less frequent: once per month, once every 3 months, or even once per year.	Three women indicated meat consumption to depend on the availability of income. One interviewee indicated her husband's role as a butcher to facilitate access to meat in their household. Two said small dried fish would be eaten when vegetables were not available.	Lack of money available to purchase meat is the primary constraint to consumption. One Sukuma woman identified the requirement to involve a butcher in the slaughter process as a deterrent, compared to the ease with which chickens could be slaughtered at home.
	Milk	Marked variation according to cattle ownership and seasonal availability. Milk was reported to be consumed infrequently or never by those not owning cattle and commonly but with seasonal variation in the frequency and volume (usually two to three times daily in the wet season) by those owning cattle.	Cow's milk is seen as a suitable alternative or supplement to human breast milk and was commonly reported as an early complementary food (boiled or added to porridge) by those with cattle. Several women indicated cow's milk would be given to young infants left in the care of others during the day while their mother was engaged in agricultural work. One mother reported milk to have been given to help her daughter grow.	Not owning cattle, a lack of funds to buy milk and limited availability for sale were identified as common barriers. For cattle‐owning households, the amount of milk was said to vary considerably between seasons, according to feed availability and the reproductive status of cows. One mother indicated the milk from a cow to vary from 200 ml per day towards the end of the dry season to 2 L in the wet season.
	Beans	Marked variation: rarely in some households, three times weekly in others. One mother indicated beans would be given preferentially to children, when available.	Enjoyed by children. One mother described adding beans to her child's porridge to promote growth, as instructed by health staff. Another said eating beans would be more common when green leafy vegetables were not available (e.g., towards the end of the dry season).	Lack of funds to purchase from local markets. Beans were not grown by any of the interviewed households. Decreased consumption was described in the previous year, when poor rainfall adversely impacted agricultural yields and household income.
Cereals and tubers 6–11 servings per day One serving is equal to a fist‐sized portion of cooked sweet potatoes.	Sweet potatoes	Marked variation between households, particularly between the two predominant language groups of the study area (Sukuma and Gogo). In Sukuma households, sweet potatoes are commonly eaten as the first meal of the day.	Both white‐ and orange‐fleshed varieties were reported to be enjoyed. One interviewee described children putting on weight at times of year when sweet potatoes are being eaten.	Not commonly grown by members of Gogo households, for the suggested reason (from members of both language groups) that the cultivation techniques are arduous, particularly without access to draught power. One Gogo mother reported sweet potatoes to cause bloating in her children.
Vegetables 3–5 servings per day One serving is equal to a palm‐sized portion of cooked vegetables.	Green leafy vegetables	Universally commonly consumed. All women reported eating green leafy vegetables on a daily or twice‐daily basis for most of the year, usually as the main (and sometimes the only) accompaniment to the staple carbohydrate.	Availability. A range of both cultivated and noncultivated green leafy vegetables are eaten. Examples given included amaranth, sweet potato leaves, jute mallow, and *kipari* (a noncultivated plant growing in the study area).	Unavailability. Green leafy vegetables are commonly harvested and dried in April, after the rains. There may be times later in the year, towards the end of the dry season, when the supply of dry leaves has been exhausted and fresh leaves are not yet available.
Fruit 2–4 servings per day One serving is equal to A single orange or banana A palm‐sized piece of watermelon or papaya	Fruit	Not commonly consumed, except for baobab fruit. Varying frequency according to households' financial capacity to purchase fruit at local markets and the seasonal availability of noncultivated fruits.	Enjoyed by children. Powder from baobab fruit was commonly reported to be added to children's porridge in the early phase of complementary feeding, together with sugar, to enhance the flavour. One mother cited fruit as being beneficial for children's growth.	Lack of disposable income for the purchase of fruit such as bananas from local markets. Wild fruits are only available at certain times of year. Two interviewees reported the sale of baobab fruit to be prioritised over home consumption, to fund the purchase of staple foods.

**Table 4:** Consumption of poultry products by enrolled children and breastfeeding women in Sanza Ward, by month of data collection (based on pictorial record charts completed by households)

	Aug 2014*	Dec 2014*	Apr 2015	Aug 2015	Dec 2015
Completed dietary records (*n*)					
Enrolled children	200	197	206	177	147
Breastfeeding women	202	192	153	83	64
All participants					
Chicken eaten at least once in month (%)					
Enrolled children	21.5	16.2	14.6	13.0	14.3
Breastfeeding women	22.8	16.7	18.3	12.0	12.5
Eggs eaten at least once in month (%)					
Enrolled children	18.0	7.1	10.7	14.8	14.3
Breastfeeding women	15.3	7.8	9.8	15.0	19.0
Participants consuming poultry products in given month					
No. meals with chicken in month, mean (*SD*)					
Enrolled children	2.3 (1.6)	1.8 (1.1)	2.3 (1.9)	2.5 (2.2)	2.6 (1.5)
Breastfeeding women	2.3 (1.6)	1.8 (1.1)	2.0 (1.8)	2.2 (3.0)	3.5 (2.5)
No. meals with eggs in month, mean (*SD*)					
Enrolled children	3.0 (2.0)	1.5 (0.6)	2.2 (1.0)	3.1 (2.7)	5.1 (5.5)
Breastfeeding women	3.1 (1.9)	1.5 (0.7)	2.3 (0.8)	3.3 (3.6)	7.7 (7.0)

*Note*. *SD* = standard deviation.

* Exclusively breastfed children excluded from analyses (six children in August 2014 and one child in December 2014).

### 3.5 Household diets

Beyond the initial phase of complementary feeding in which sweetened cereal‐based porridges predominate, there was consensus among interviewees that children's diets would increasingly reflect the foods eaten by other members of their household. It was reported that by 24 months of age, there would be no appreciable differences between children's and adults' diets. Table 3 highlights the variation in consumption frequency for selected food items between households, compiled from information provided during in‐depth interviews, together with reported reasons for eating certain foods and barriers to their more frequent inclusion in household diets. Consumption of beans and meat from cattle or small ruminants was reported to be reliant on households having disposable income for their purchase. Such items were reported to be eaten several times per week in some households and very occasionally, perhaps even only once per year, in others. Consumption of poultry products was indicated to be closely associated with ownership of chickens and on the number of chickens owned. As discussed further in the next section, for many households, particularly those with small flocks, eating chicken was very rare and often reserved for festive occasions, visiting guests or occasions when birds had died of disease.

Only seven women (17.9%) reported having made any conscious changes in their diet while breastfeeding. Cereal‐based porridge was the most common food item reported to promote milk production, sometimes with the addition of sugar or groundnuts: “When you have children, you should drink porridge, and then the milk will come” (24‐year‐old woman from Chicheho Village, Sanza Ward). Several women said larger portions of their regular diet would be eaten, but it was noted that pain or poor appetite in the early post‐partum period might make this difficult. One interviewee expressed exasperation at the notion that a different diet might be achieved in the local setting: “You can see the difficult situation here. How could I eat any special foods?” (woman of unknown age from Mpandagani Village, Majiri Ward). Another described seasonal variation in food availability as being significant: “When there is rainfall, there are a lot of (green leafy) vegetables. You can mix them with tomatoes and other vegetables. It is easier to breastfeed in the wet season” (34‐year‐old woman from Sanza Village, Sanza Ward).

### 3.6 Consumption of poultry products

Qualitative data exploring the contributions of chicken meat and eggs to local diets indicate consumption frequency to vary substantially within the study area, as outlined in Table 3. Of eight interviewees asked about their motivations for keeping chickens, all cited opportunities for income generation—to meet small household expenses, school fees, children's clothing, and medical costs—as a primary reason, two mentioned their use as gifts for visitors, and one spoke of direct contributions to household diets in times of food scarcity. “When you have a problem getting vegetables, you can slaughter a chicken or even eat some eggs” (52‐year‐old woman, Ikasi Village, Sanza Ward).

A common theme emerging from interviews was the low frequency with which poultry products were consumed in a majority of households: constrained by limited availability and, among chicken‐owning households, a sense of the need to retain chickens to sell in times of need, and eggs to hatch to replace bird losses. Household size was identified by one interviewee, living with her husband, nine children, three of her children's spouses and six grandchildren, as an important factor: “If there is a couple and one child, it is easy to eat (chicken). Even if I have a lot of chickens, I still have a big family to feed” (woman of unknown age from Mahaka Village, Majiri Ward).

Analysis of completed dietary record charts from Sanza Ward was undertaken for children enrolled in the longitudinal study and, where present, for a breastfeeding woman within the same household, typically the child's mother. Records for pregnant women were excluded from this analysis, due to low numbers within the study population (in which all households included a child under 24 months of age at the time of enrolment), and confusion in some instances over how to record the absence of a pregnant woman, as distinct from a pregnant woman being present but not consuming any poultry products. Questionnaire data on the timing of the introduction of complementary feeding were used to identify exclusively breastfed children at the time of dietary records being completed, enabling them to be omitted from analyses of chicken and egg consumption. This resulted in exclusion of six children in the first application of the research tool and one child in the second application.

Fluctuation in the number of completed records was seen due to some households travelling at the time of data collection, relocating outside the study area, or choosing not to continue to participate. A substantial decrease in the number of completed records for breastfeeding women can be noted with increasing time, as children enrolled in the study were weaned. Across all months of data collection, very low levels of consumption of chicken meat and eggs were recorded among both young children and breastfeeding women. Table 4 shows the percentage of women and children eating poultry products at least once during each month‐long period of data collection—in the case of children consuming meals containing chicken, ranging from 12.5% to 21.8% (in August 2015 and August 2014, respectively). For both chicken and eggs, household‐level records indicate that a large majority of women and children do not consume these products even once over the course of a given month.

The probability of consuming chicken meat and eggs differed significantly between data collection periods (both *p* < .001), as assessed using separate binomial generalised linear mixed models allowing for geographic clustering. There is no indication of a seasonal pattern based on this limited period of data collection. In addition to a low proportion of women and children consuming chicken and eggs, the frequency with which these products were consumed was also very low. Among those participants reported to have consumed chicken, a mean of 1.8–2.6 meals per month was recorded for children and 1.8–3.5 for breastfeeding mothers. Egg consumption over a period of 1 month was similarly uncommon (7.1–18.0% of children and 7.8–19.0% breastfeeding women) and infrequent (1.5–5.1 meals per month for children and 1.5–7.7 meals per month for breastfeeding women, among those consuming eggs in a given month).

**Figure 4:**
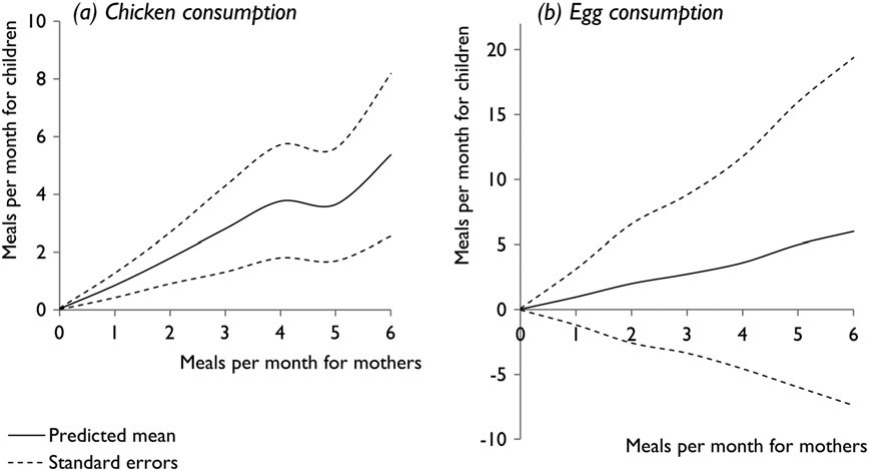
Predicted number of meals containing poultry products consumed by children per month, according to maternal consumption frequency (based on pictorial record charts completed by households)

Poisson generalised linear mixed models were used to test the significance of the number of meals containing poultry products consumed by mothers as a predictor for the number consumed by their children. Chicken meat and eggs were evaluated through separate models. For both chicken meat and eggs, the number of meals consumed by a woman in a given month was significantly positively associated (both *p* < .001) with the number consumed by her child in the same month. The predicted consumption frequency of poultry products by a child rose with increasing consumption frequency by their mother (see Figure 4 and Table 5). Large standard errors were noted to be associated with model‐based means for egg consumption within this population, where eggs are very infrequently eaten. Summary output of regression coefficients and variance components from both models is shown in Table 5.

**Table 5:** Regression coefficients and variance components from Poisson generalised linear mixed models indicating the number of meals containing poultry products consumed by a mother to be a significant predictor for the number consumed by her child. Separate models have been used for (a) chicken meat and (b) eggs

(a) Chicken (*p* < .001)				(b) Eggs (*p* < .001)			
Fixed effect		Coeff	*SE*	Fixed effect		Coeff	*SE*
Constant		−3.153	0.516	Constant		−3.528	0.469
No. of meals per month consumed by child's mother	0	0	—	No. of meals per month consumed by child's mother	0	0	
	1	2.995	0.250		1	3.494	0.295
	2	3.738	0.237		2	4.221	0.276
	3	4.185	0.307		3	4.531	0.293
	4	4.478	0.292		4	4.809	0.284
	5	4.447	0.318		5	5.137	0.304
	6	4.834	0.298		6	5.32	0.293
	7	5.169	0.473		7	5.474	0.442
	8	—	—		8	5.607	0.339
	9	5.496	0.459		12	5.648	0.304
	10	5.345	0.394		14	6.167	0.352
					25	6.706	0.307

Random effect		Variance	*SE*	Random effect		Variance	*SE*
Ward		0.218	—	Ward		0.167	—
Ward and village		0	—	Ward and village		0	—
Ward, village, and subvillage		0.109	0.089	Ward, village, and subvillage		0	—
Ward, village, subvillage, and ID		0	—	Ward, village, subvillage, and ID		0	—
Dispersion		1	—	Dispersion		1	—

*Note*. *SE* = standard error; Coeff = coefficient.

No significant association was identified between child age and the consumption of either chicken or eggs. During focus group discussions conducted within the overarching research project, in which tea and hard‐boiled eggs were served to participants, four women were observed on separate occasions to take small pieces of the yolk, mould it to a smooth shape, and feed it to their young children. On questioning, women indicated the softer texture of the yolk to be more suitable and palatable for infants. Five women acknowledged traditional beliefs within the area precluding the consumption of eggs by male children prior to circumcision, at around 2 years of age. Four indicated this to be a practice followed within their own families, justified by fears about interference with wound healing, eggs appearing at the preputial opening following circumcision and family customs. Four other interviewed women denied the existence of any food customs related to children's gender. Univariable analysis was suggestive of female children consuming eggs more frequently than male children (*p* = .07), but this was not significant when combined with mothers' egg consumption within a multivariable model (*p* = .20).

## 4 DISCUSSION

Infant and young child feeding is an inherently complex area of research, whereby care‐giving behaviours reflect an interplay of social, cultural, economic, and environmental influences. Among women living in affluent countries, including African refugee populations, breastfeeding difficulties and a departure from intended child feeding practices have been associated with maternal discontent and feelings of culpability (Burns, Schmied, Sheehan, & Fenwick, 2010; Hufton & Raven, 2016; Murphy, 1999). Where child feeding practices deviate from guidelines provided by health personnel, mothers may be unwilling to speak openly and candidly about their experiences. There is potential for added complexity when working with low‐literacy and low‐income populations, in which a heightened power imbalance often exists between breastfeeding women and those working in the health sector (Bassett, Bijlmakers, & Sanders, 1997; Molyneux, Peshu, & Marsh, 2005).

Against this backdrop, researchers seeking to document IYCF practices may be faced with further challenges of cultural and linguistic differences, perceived hierarchies between investigators and participants, and an unfamiliarity with processes of data collection and informed consent (Molyneux, Wassenaar, Peshu, & Marsh, 2005). Among limitations of this study, the potential for acquiescence bias was recognised, whereby participants tend to respond positively to neutral questions, as well as social desirability bias, where answers that are perceived to be more acceptable than true attitudes or behaviours may be given (Kaminska & Foulsham, 2013; Ross & Mirowsky, 1984). A recent study in Central America has suggested that a physical inability to breastfeed might be considered an acceptable justification for early initiation of complementary feeding but that women may be unwilling to discuss reasons for not wanting to breastfeed (Safon et al., 2017). By identifying herself with her disciplinary background as a veterinarian, the primary investigator in the present study sought to minimise association with medical personnel, with whom nonadherence to IYCF guidelines has been suggested to be less likely to be shared (Mabilia, 2005).

Widespread familiarity with recommendations for exclusive breastfeeding to 6 months was evident among study participants, and “6 months” was a common initial response to interview questions about the timing of initiation of complementary feeding. With further probing, clarification about specific food items and reference to the experiences of other mothers within the community, it was common for interviewees to proceed to disclose specific breastfeeding challenges and to amend their response to indicate a younger age of complementary feeding. Some uncertainty surrounds the level of accuracy that might be achieved by enumerators, documenting similar information through structured questions within the context of a multipage survey.

Motivations for early commencement of complementary feeding provide an example of the value of an explanatory sequential mixed methods approach. Analysis of questionnaire‐based data identified inadequate breast milk as the primary factor influencing decision‐making for a substantial majority (95.0%) of mothers. This response encompasses the broad and complex phenomenon of perceived insufficient milk supply and served to highlight an area for further exploration through qualitative methods. In‐depth interviews revealed persistent crying and fractious behaviour as the predominant triggers for women to deem their breast milk inadequate—in quantity or quality—to meet their child's nutritional needs. The recurring notion of “watery” breast milk aligns with previous reports of Tanzanian women viewing consistency as an important indicator of milk quality (Mabilia, 2005) and warrants closer investigation to understand against which reference this judgement is made.

Insufficient milk supply was noted to have been a recurrent experience for several multiparous women. References to previous lactation experiences and, in one case, those of an interviewee's own mother conveyed a sense of the capacity to breastfeed being intrinsic to an individual—perhaps with familial influences. Beyond an increase in the consumption of cereal‐based porridges, dietary changes among breastfeeding women appear infrequent. A marked disparity is evident between existing diets and the diversity of food groups advocated in government‐endorsed extension materials (Ministry of Health and Social Welfare, 2012), which depict a marked departure from the monotonous cereal‐based diets common among this population and which would necessitate a substantial shift in the allocation of household resources.

Although this paper highlights early initiation of complementary feeding as the common deviation from WHO recommendations in this setting, a small minority (3.2%) of the study sample reported having continued exclusive breastfeeding beyond 6 months of age. During interviews, two of these women conveyed a sense of accomplishment—that for their child, their breast milk was “enough.” It is noted that beyond 6 months of age, low levels of iron and zinc in breast milk and depletion of prenatal stores place exclusively breastfed infants at risk of deficiency if an exogenous source of these micronutrients is not provided (Butte, Lopez‐Alarcon, & Garza, 2002; Dewey, 2013). It has also been proposed that many mothers are unable to meet the energy requirements of a 6‐month‐old infant, based on the metabolisable energy content of breast milk and the quantity of milk transfer at this time (Reilly & Wells, 2005).

Cereal‐based porridges were identified as the first semi‐solid food across the entire study sample, described during interviews as a watery gruel suited to infants' limited swallowing ability. Capturing information about the range and timing of introduction of specific complementary foods proved difficult, both in survey questions and in retrospective discussions with mothers. Although some interviewees were forthcoming with specific information about their child's dietary patterns and food preferences, others elaborated little on the topic. Although questionnaire responses indicate groundnuts, beans, and baobab fruit to be among the foods added to infants' porridge, in‐depth interview data suggest limited access to these items for many families.

Household diets varied substantially across the community. Where barriers to the consumption of specific foods were widespread across the study population, such as the seasonal availability of green leafy vegetables, little variation was seen in the consumption frequency reported by women. Where barriers relate to traditions, household size, livestock ownership, or income availability, larger differences in consumption frequency appear to emerge. Milk consumption was one such food item: consumed daily in many cattle‐owning households and very rarely in households without cattle. As for complementary feeding practices, it proved difficult to estimate the quantity of cow's milk consumed within the household, and how this might vary with seasonal changes in feed availability for cattle.

Although previous research in Tanzania documented an increase in the number of chickens and eggs sold, bartered, or consumed over a 3‐year period following the introduction of Newcastle disease vaccination (Harun et al., 2009), pictorial record charts in this study indicated infrequent consumption of poultry products across all data collection periods. This is not surprising in the early stages of establishment of Newcastle disease control programs in the study area and in the face of increasing weather variability. Community members' long‐term experience of seasonal disease outbreaks and high levels of mortality in free‐ranging chicken flocks has been linked to infrequent consumption of eggs and chickens, with poultry keepers prioritising the hatching of eggs to provide replacement stock and retention of chickens for sale in times of financial need (Alders & Pym, 2009; Bagnol, 2001; Pym, Guerne Bleich, & Hoffman, 2006)—as attested by interviewed mothers within this area.

These record charts appear to have proved effective as an approach to household‐level data collection; however, it should be acknowledged that their use was reliant on the involvement of Community Assistants employed in each village. These representatives have been responsible for training representatives from each household to complete the charts and overseeing their progress through the month‐long data collection period. Despite a subjectively simple design, the process of identifying the appropriate location within a table layout to record a particular meal consumed by a particular household member was not intuitive for all participants, in communities where almost one third of women (32.5%) have had no access to formal education.

Despite very low consumption of chicken meat and eggs within this population, the finding of close associations between mothers and their children eating these items is promising. Documentation of these dietary patterns from five 1‐month records over a 20‐month period provides encouragement that programs that increase the consumption of poultry products at a household level will bring direct nutritional benefits for infants and young children within those households—an outcome that would be expected to be enhanced through targeted awareness‐raising activities relating to child nutrition. Within many cultures, traditional beliefs, and taboos surround the eating of eggs by young children and pregnant women (Iannotti & Roy, 2013; Meyer‐Rochow, 2009; Trant, 1954). In this study, qualitative data identified customs precluding the consumption of eggs by uncircumcised male children but appeared to be variably practised across the community and dietary records showed no significant gender‐based differences in egg consumption frequency.

## 5 CONCLUSIONS

In a setting where caring for children is an almost universally held role for women and the intergenerational transfer of care‐giving information begins at a young age, questions by outsiders about breastfeeding and complementary feeding practices can appear senseless and may trigger suspicion in many rural African communities. The use of a mixed methods approach in this study sought to facilitate triangulation of findings, reflect on methodological approaches better suited to specific topics, and use targeted discussions with individual informants to more deeply explore findings from sample‐wide questionnaires. Low and abnormally timed rainfall during the study period resulted in an unforeseen level of mobility among participating households, with a drastically reduced harvest prompting some to relocate outside the area to pursue alternative livelihood strategies. With increasing weather variability in the future, studies of populations reliant on rain‐fed agriculture should consider the potential of increased participant dropout and adjust sample size calculations accordingly.

As shown at a national level in Tanzania (Ministry of Health, Community Development, Gender, Elderly and Children, Ministry of Health, National Bureau of Statistics, Office of the Chief Government Statistician and ICF, 2016), breastfeeding was confirmed to be widespread and of long duration in these rural communities in Manyoni District. However, with 74.4% of mothers commencing complementary feeding prior to 6 months—primarily motivated by maternal perceptions of insufficient milk supply—it is clear that guidelines intended to maximise the likelihood of infants' nutritional needs being met are falling short of their potential impact. Although women within the study described receiving nutritional information of a general nature during routine perinatal visits to local health facilities, older female family members were identified as the usual source of guidance for specific challenges. Conflict is evident between advice from these sources for early complementary feeding as a response to breastfeeding difficulties and nationally endorsed IYCF recommendations, which advocate exclusive breastfeeding to 6 months but may fail to address specific challenges using culturally sensitive approaches.

Within programs seeking to influence diets through improved access to nutrient‐rich foods, an understanding of local beliefs, practices, constraints, and priorities is key to achieving impact. This study highlights the importance of involving both local health staff and broader family networks in addressing maternal perceptions of inadequate breast milk production. Chicken meat and eggs were rarely eaten among study participants, yet children's intake was shown to follow their mothers', and no significant gender‐based barriers to consumption by children were found. As poultry health programs reduce mortality and increase chicken ownership and flock size within these communities, families will be faced with decisions about the management and use of poultry. Culturally appropriate messaging should acknowledge the multiple contributions of chickens, as an accessible income source and a means of participating in traditional customs, while promoting their direct nutritional benefits for young children and women of reproductive age.

## CONFLICTS OF INTEREST

The authors declare that they have no conflicts of interest.

## CONTRIBUTIONS

JDB developed the primary concept of the paper; BB, IDH, RA, and WM provided input into development of the qualitative component of the study; JDB conducted in‐depth interviews and led data analysis; PT provided support for quantitative analyses and BB for qualitative analyses; JDB wrote the initial and subsequent drafts of the paper; and BB, IDH, PT, RA, and WM contributed to critical revision of the manuscript.

## References

[mcn12550-bib-0001] Alders, R. G. (2014). Making Newcastle disease vaccines available at village level. Veterinary Record, 174(20), 502–503. 24832887 10.1136/vr.g3209

[mcn12550-bib-0002] Alders, R. , Aongolo, A. , Bagnol, B. , de Bruyn, J. , Kimboka, S. , Kock, R. , … Young, M. (2014). Using a one health approach to promote food and nutrition security in Tanzania and Zambia. GRF Davos Planet@Risk Special Issue on One Health, 2, 187–190.

[mcn12550-bib-0003] Alders, R. G. , & Pym, R. A. E. (2009). Village poultry: Still important to millions, eight thousand years after domestication. World's Poultry Science Journal, 65(2), 181–190.

[mcn12550-bib-0004] Arts, M. , Geelhoed, D. , De Schacht, C. , Prosser, W. , Alons, C. , & Pedro, A. (2011). Knowledge, beliefs, and practices regarding exclusive breastfeeding of infants younger than 6 months in Mozambique: A qualitative study. Journal of Human Lactation, 27(1), 25–32. 21177988 10.1177/0890334410390039

[mcn12550-bib-0005] Bagnol, B. (2001). The social impact of Newcastle disease control. Paper presented at the international workshop on Newcastle disease control in village chickens, Maputo, Mozambique. March 6-9, 2000

[mcn12550-bib-0006] Bassett, M. T. , Bijlmakers, L. , & Sanders, D. M. (1997). Professionalism, patient satisfaction and quality of health care: Experience during Zimbabwe's structural adjustment programme. Social Science & Medicine, 45(12), 1845–1852. 9447633 10.1016/s0277-9536(97)00122-6

[mcn12550-bib-0007] Black, M. M. (2003). Micronutrient deficiencies and cognitive functioning. Journal of Nutrition, 133(11 Suppl 2), 3927S–3931S. 14672291 10.1093/jn/133.11.3927S PMC3140638

[mcn12550-bib-0008] Black, R. , Victora, C. , Walker, S. , Bhutta, Z. , Christian, P. , de Onis, M. , … Uauy, R. (2013). Maternal and child undernutrition and overweight in low‐income and middle‐income countries. Lancet, 382(9890), 427–451. 23746772 10.1016/S0140-6736(13)60937-X

[mcn12550-bib-0009] Burns, E. , Schmied, V. , Sheehan, A. , & Fenwick, J. (2010). A meta‐ethnographic synthesis of women's experience of breastfeeding. Maternal & Child Nutrition, 6(3), 201–219. 20929493 10.1111/j.1740-8709.2009.00209.x PMC6860551

[mcn12550-bib-0010] Butte, N. , Lopez‐Alarcon, M. , & Garza, C. (2002). Nutrient adequacy of exclusive breastfeeding for the term infant during the first six months of life. Geneva, Switzerland: World Health Organization.

[mcn12550-bib-0011] Buxton, K. E. , Gielen, A. C. , Faden, R. R. , Brown, C. H. , Paige, D. M. , & Chwalow, A. J. (1991). Women intending to breastfeed: Predictors of early infant feeding experiences. American Journal of Preventive Medicine, 7(2), 101–106. 1910883

[mcn12550-bib-0012] Covarrubias, K. , Nsiima, L. , & Zezza, A. (2012). Livestock and livelihoods in rural Tanzania: A descriptive analysis of the 2009 National Panel Survey, Joint paper of the World Bank, FAO, AU‐IBAR, ILRI and the Tanzania Ministry of Livestock and Fisheries Development, July 2012.

[mcn12550-bib-0013] Dettwyler, K. A. , & Fishman, C. (1992). Infant feeding practices and growth. Annual Review of Anthropology, 21, 171–204.

[mcn12550-bib-0014] Dewey, K. G. (2013). The challenge of meeting nutrient needs of infants and young children during the period of complementary feeding: An evolutionary perspective. The Journal of Nutrition, 143(12), 2050–2054. 24132575 10.3945/jn.113.182527 PMC3827643

[mcn12550-bib-0015] Fabrizio, C. S. , van Liere, M. , & Pelto, G. (2014). Identifying determinants of effective complementary feeding behaviour change interventions in developing countries. Maternal & Child Nutrition, 10(4), 575–592. 24798264 10.1111/mcn.12119 PMC4282339

[mcn12550-bib-0016] Francis, S. C. , Baisley, K. , Lees, S. S. , Andrew, B. , Zalwango, F. , Seeley, J. , … Hayes, R. J. (2013). Vaginal practices among women at high risk of HIV infection in Uganda and Tanzania: Recorded behaviour from a daily pictorial diary. PLoS One, 8(3). e59085 10.1371/journal.pone.0059085 PMC3608607 23555618

[mcn12550-bib-0017] Francis, S. C. , Lees, S. S. , Andrew, B. , Zalwango, F. , Vandepitte, J. , Ao, T. , … Hayes, R. (2012). Vaginal practices diary: Development of a pictorial data collection tool for sensitive behavioral data. Sexually Transmitted Diseases, 39(8), 614–621. 22801344 10.1097/OLQ.0b013e3182515fe4 PMC3827744

[mcn12550-bib-0018] Greiner, T. , van Esterik, P. , & Latham, M. C. (1981). The insufficient milk syndrome: An alternative explanation. Medical Anthropology, 5(2), 233–247.

[mcn12550-bib-0019] Gussler, J. D. , & Briesemeister, L. H. (1980). The insufficient milk syndrome: A biocultural explanation. Medical Anthropology, 4(2), 145–174.

[mcn12550-bib-0020] Harun, M. , Alders, R. G. , Sprowles, L. , Bagnol, B. , Cambaza, A. B. , Msami, H. , & Mgomezulu, R. (2009). Southern Africa Newcastle Disease Control Project impact studies: Baseline and participatory rural appraisal results. *Village chickens, poverty alleviation and the sustainable control of Newcastle disease: Proceedings of an international conference*, Dar es Salaam, Tanzania, 5–7 October 2005.

[mcn12550-bib-0021] Hill, P. D. , & Aldag, J. (1991). Potential indicators of insufficient milk supply syndrome. Research in Nursing & Health, 14(1), 11–19. 2017578 10.1002/nur.4770140104

[mcn12550-bib-0022] Hufton, E. , & Raven, J. (2016). Exploring the infant feeding practices of immigrant women in the North West of England: A case study of asylum seekers and refugees in Liverpool and Manchester. Maternal & Child Nutrition, 12(2), 299–313. 25243979 10.1111/mcn.12145 PMC6860086

[mcn12550-bib-0023] Iannotti, L. L. , Lutter, C. K. , Stewart, C. P. , Gallegos Riofrío, C. A. , Malo, C. , Reinhart, G. , … Waters, W. F. (2017). Eggs in early complementary feeding and child growth: A randomized controlled trial. Pediatrics. 10.1542/peds.2016-3459 28588101

[mcn12550-bib-0024] Iannotti, L. , & Roy, D. (2013). Nutritional impact of highly pathogenic avian influenza in Kenya. Food and Nutrition Bulletin, 34(3), 299–309. 24167910 10.1177/156482651303400302

[mcn12550-bib-0025] Jones, G. , Steketee, R. W. , Black, R. E. , Bhutta, Z. A. , & Morris, S. S. (2003). How many child deaths can we prevent this year? Lancet, 362(9377), 65–71. 12853204 10.1016/S0140-6736(03)13811-1

[mcn12550-bib-0026] Kaminska, O. , & Foulsham, T. (2013). Understanding sources of social desirability bias in different modes: Evidence from eye‐tracking. Retrieved from https://www.iser.essex.ac.uk/research/publications/working-papers/iser/2013-04.pdf

[mcn12550-bib-0027] Kubik, Z. , & Maurel, M. (2016). Weather shocks, agricultural production and migration: Evidence from Tanzania. The Journal of Development Studies, 52, 5,665–5,680. 10.1080/00220388.2015.1107049

[mcn12550-bib-0028] Lema, M. , & Majule, A. E. (2009). Impacts of climate change, variability and adaptation strategies on agriculture in semi arid areas of Tanzania: the case of Manyoni District in Singida Region, Tanzania. African Journal of Environmental Science and Technology, 3(8), 206–218.

[mcn12550-bib-0029] Mabilia, M. (2005). Breast feeding and sexuality: Behaviour, beliefs and taboos among the Gogo mothers in Tanzania. New York: Berghahn Books.

[mcn12550-bib-0030] Meyer‐Rochow, V. B. (2009). Food taboos: Their origins and purposes. Journal of Ethnobiology and Ethnomedicine, 5(1), 18. 19563636 10.1186/1746-4269-5-18 PMC2711054

[mcn12550-bib-1005] Ministry of Health and Social Welfare . (2012). *Lishe wakati wa* *ujauzito na kunyonyesha* . [Nutrition during pregnancy and breastfeeding]. [Pamphlet].

[mcn12550-bib-0031] Ministry of Health, Community Development, Gender, Elderly and Children [Tanzania Mainland] , Ministry of Health [Zanzibar] , National Bureau of Statistics , Office of the Chief Government Statistician and ICF . (2016). Tanzania Demographic and Health Survey and Malaria Indicator Survey 2015–16. Dar es Salaam, Tanzania and Rockville, Maryland, USA.

[mcn12550-bib-0032] Molyneux, C. S. , Peshu, N. , & Marsh, K. (2005). Trust and informed consent: Insights from community members on the Kenyan coast. Social Science & Medicine, 61(7), 1463–1473. 16005781 10.1016/j.socscimed.2004.11.073

[mcn12550-bib-0033] Molyneux, C. S. , Wassenaar, D. R. , Peshu, N. , & Marsh, K. (2005). “Even if they ask you to stand by a tree all day, you will have to do it (laughter)…!”: Community voices on the notion and practice of informed consent for biomedical research in developing countries. Social Science & Medicine, 61(2), 443–454. 15893058 10.1016/j.socscimed.2004.12.003

[mcn12550-bib-0034] Murphy, E. (1999). “Breast is best”: Infant feeding decisions and maternal deviance. Sociology of Health & Illness, 21(2), 187–208.

[mcn12550-bib-0035] Neumann, C. G. , Murphy, S. P. , Gewa, C. , Grillenberger, M. , & Bwibo, N. O. (2007). Meat supplementation improves growth, cognitive, and behavioral outcomes in Kenyan children. Journal of Nutrition, 137(4), 1119–1123. 17374691 10.1093/jn/137.4.1119

[mcn12550-bib-0036] Pym, R. A. E. , Guerne Bleich, E. , & Hoffman, I. (2006). The relative contribution of indigenous chicken breeds to poultry meat and egg production and consumption in the developing countries of Africa and Asia. Paper presented at the XII European Poultry Conference, Verona, Italy.

[mcn12550-bib-0037] Reilly, J. J. , & Wells, J. C. (2005). Duration of exclusive breast‐feeding: Introduction of complementary feeding may be necessary before 6 months of age. British Journal of Nutrition, 94(6), 869–872. 16351760 10.1079/bjn20051601

[mcn12550-bib-0038] Ross, C. E. , & Mirowsky, J. (1984). Socially‐desirable response and acquiescence in a cross‐cultural survey of mental health. Journal of Health and Social Behavior, 189–197. 6470459

[mcn12550-bib-0039] Sacco, L. M. , Caulfield, L. E. , Gittelsohn, J. , & Martínez, H. (2006). The conceptualization of perceived insufficient milk among Mexican mothers. Journal of Human Lactation, 22(3), 277–286. 16885488 10.1177/0890334406287817

[mcn12550-bib-0040] Safon, C. , Keene, D. , Guevara, W. J. U. , Kiani, S. , Herkert, D. , Muñoz, E. E. , & Pérez‐Escamilla, R. (2017). Determinants of perceived insufficient milk among new mothers in León, Nicaragua. Maternal & Child Nutrition, 13(3). e12369 10.1111/mcn.12369 PMC6865952 27650889

[mcn12550-bib-0041] Segura‐Millan, S. , Dewey, K. G. , & Perez‐Escamilla, R. (1994). Factors associated with perceived insufficient milk in a low‐income urban population in Mexico. Journal of Nutrition, 124(2), 202–212. 8308569 10.1093/jn/124.2.202

[mcn12550-bib-0042] Tanzania Food and Nutrition Centre . (2011). Zuia magonjwa sugu yasiyo ya kuambukiza kwa kuzingatia ulaji unaofaa na mtindo bora wa maisha [Prevention of chronic non‐communicable disease through balanced food intake and improved lifestyle].

[mcn12550-bib-0043] Trant, H. (1954). Food taboos in East Africa. The Lancet, 264(6840), 703–705. 10.1016/s0140-6736(54)90471-7 13202477

[mcn12550-bib-0044] Tully, J. , & Dewey, K. G. (1985). Private fears, global loss: A cross‐cultural study of the insufficient milk syndrome. Medical Anthropology, 9(3), 225–243. 3842148 10.1080/01459740.1985.9965934

[mcn12550-bib-0045] Turk, J. (2013). Poverty, livestock and food security in developing countries. CAB Reviews, 8(33), 1–8.

[mcn12550-bib-0046] Victor, R. , Baines, S. K. , Agho, K. E. , & Dibley, M. J. (2014). Factors associated with inappropriate complementary feeding practices among children aged 6–23 months in Tanzania. Maternal & Child Nutrition, 10(4), 545–561. 22925557 10.1111/j.1740-8709.2012.00435.x PMC6860229

[mcn12550-bib-0047] World Health Organization . (2002). Infant and young child nutrition: Global strategy on infant and young child feeding (A55/15).

[mcn12550-bib-0048] World Health Organization . (2006). WHO Child Growth Standards. Length/height‐for‐age, weight‐for‐age, weight‐for‐length, weight‐for‐height and body mass index‐for‐age: Methods and development.

